# Magnetic Anisotropy and Damping Constant of Ferrimagnetic GdCo Alloy near Compensation Point

**DOI:** 10.3390/ma14102604

**Published:** 2021-05-17

**Authors:** Sungjung Joo, Rekikua Sahilu Alemayehu, Jong-Guk Choi, Byong-Guk Park, Gyung-Min Choi

**Affiliations:** 1 Center for Electromagnetic Metrology, KRISS, Daejeon 34113, Korea; joosj@kriss.re.kr; 2 Department of Energy Science, Sungkyunkwan University, Suwon 16419, Korea; rekiks2@gmail.com; 3 Department of Materials Science and Engineering, KAIST, Daejeon 34141, Korea; cjg000@kaist.ac.kr (J.-G.C.); bgpark@kaist.ac.kr (B.-G.P.); 4 Center for Integrated Nanostructure Physics, Institute for Basic Science (IBS), Suwon 16419, Korea

**Keywords:** ferrimagnet, GdCo alloy, magnetic anisotropy, damping constant

## Abstract

Metallic ferrimagnets with rare earth-transition metal alloys can provide novel properties that cannot be obtained using conventional ferromagnets. Recently, the compensation point of ferrimagnets, where the net magnetization or net angular momentum vanishes, has been considered a key aspect for memory device applications. For such applications, the magnetic anisotropy energy and damping constant are crucial. In this study, we investigate the magnetic anisotropy and damping constant of a GdCo alloy, with a Gd concentration of 12–27%. By analyzing the equilibrium tilting of magnetization as a function of the applied magnetic field, we estimate the uniaxial anisotropy to be 1–3 × 10^4^ J m^−3^. By analyzing the transient dynamics of magnetization as a function of time, we estimate the damping constant to be 0.08–0.22.

## 1. Introduction

Rare earth (RE)-transition metal (TM) alloys can have a ferrimagnetic phase, where the magnetizations of the RE and TM sublattices have antiparallel alignment. RE-TM ferrimagnets possess many interesting properties; e.g., perpendicular magnetic anisotropy without long-range crystalline ordering [1,2,3,4], all-optical switching of magnetization [5,6], net magnetization vanishing at the compensation point [7,8]. Recently, RE-TM ferrimagnets have been considered as information-storage elements for memory devices of magnetic-random access memory and race track memory [9,10,11,12,13,14,15]. An advantage of RE-TM ferrimagnets over conventional TM ferromagnets in terms of operation speed and power consumption has been demonstrated near the compensation point, where the magnetization or angular momentum of RE and TM cancel each other out. To gain more insights into the application of RE-TM ferrimagnets to memory devices, additional information regarding magnetic anisotropy and damping is required. The magnetic anisotropy determines the thermal stability of the memory element, and the damping constant determines the switching current in the writing process [16,17,18].

In this study, we investigate the magnetic anisotropy and damping of a GdCo alloy, with a Gd concentration of 12–27%. The GdCo alloy is a common material that has been used for memory devices owing to its ability to tune the net magnetization and perpendicular magnetic anisotropy. To investigate magnetic anisotropy, we measure the out-of-plane magnetization as a function of the in-plane magnetic field. Analyzing the equilibrium tilting of the magnetization, we determine the uniaxial magnetic anisotropy of 2.8 × 10^4^ J m^−3^ at the compensation point. To investigate damping, we measure the transient dynamics of magnetization, triggered by sudden change of anisotropy energy. Analyzing the relaxation of the magnetization precession, we determine the enhanced damping of 0.22 at the compensation point.

## 2. Materials and Methods

We fabricate samples of Ta (3)/Pt (5)/GdCo (10)/Ta (3) structures using magnetron sputtering on thermally oxidized silicon substrates; the numbers in the parentheses are in nm. The Ta/Pt layer acts as an underlayer of the GdCo alloy. Although the GdCo alloy has an amorphous structure without a structural phase, the underlayer affects the magnetic anisotropy of the GdCo alloy. We find that the GdCo alloy with the Ta/Pt underlayer provides a larger magnetic anisotropy than without underlayer. The exact mechanism is not known, but experimental observation of the underlayer effect has been reported with amorphous ferrimagnets of GdTbCo and TbFeCo alloys [19,20]. In this work, we fix the underlayer and vary the Gd concentration from 12% to 27% of the GdCo layer. The GdCo composition is adjusted by controlling the sputter power of the Gd and Co targets during co-sputtering. Since the deposition rate of each target is approximately proportional to the sputter power, we can control the flux of Gd and Co atoms on the substrate during the deposition. The actual composition of the GdCo film was check by Energy–dispersive X-ray Spectroscopy. The top Ta layer acts as a capping layer to protect the GdCo layer from oxidation. Regarding sputtering conditions, we use the Ar pressure of 3 mTorr, target–to–substrate distance of 200 mm, and deposition rate of 0.06–0.12 nm s^−1^. The deposition rate of each layer is predetermined with a single thick layer, whose thickness is determined by X-ray reflectivity measurements, then the thickness of each layer is controlled by deposition time. All layers are deposited at room temperature.

We investigate magnetic anisotropy using the generalized Sucksmith-Tompson (GST) method [21,22,23]. The GST method analyzes the out-of-plane magnetization in an oblique magnetic field, a combination of the in-plane field to tilt magnetization and the out-of-plane field to maintain a single domain. To accurately quantify the out-of-plane magnetization, we use a vibrating sample magnetometer (VSM) and the anomalous Hall effect (AHE). The VSM measures the stray field from magnetization and enables quantification of the absolute magnitude of magnetization. The AHE measures the anomalous voltage (*V*_AHE_) in the presence of the charge current (*I*_c_) and magnetization (*M*) as ${\vec{V}}_{\mathrm{AHE}}\propto {\vec{I}}_{c}\times \vec{M}$ [24]. When *I*_c_ and *M* are along the *x* and *z* directions, respectively, *V*_AHE_ is along the *y* direction. Because the AHE originates from the electrons near the Fermi level, it does not provide the absolute magnitude of magnetization but accurately measures the relative magnitude of the out-of-plane magnetization, $m_{z}=M_{z}/\left|M\right|$, because of the large signal-to-noise ratio of *V*_AHE_. We connect wire bonding at the four corners of the square-shaped sample, size of 1.27 × 12.7 cm^2^, to measure *V*_AHE_ in the van der Pauw geometry. We measure *V*_AHE_, with a DC charge current of 1 mA, as a function of the magnetic field with an oblique angle in the *z*-direction.

We investigate damping constants using time-resolved magneto-optical Kerr effect (TRMOKE). The TRMOKE is based on an optical pump-probe technique; i.e., a pump pulse excites the sample, and a probe pulse measures the response of the sample with a controlled time delay between the pump and probe. When a pump pulse causes a sudden change in the magnetic anisotropy, a precessional motion of the magnetization occurs [25]. A probe pulse measures the magnetization dynamics via MOKE. We use a polar MOKE geometry, so that the probe pulse measures the *z* component of the magnetization. We use a Ti-sapphire femtosecond laser to produce the pump and probe pulses. The wavelengths of the pump and probe are 784 nm. The pulse widths are 1.1 ps for the pump and 0.2 ps for the probe. (The pump pulse is elongated by a group velocity dispersion of the electro-optic modulator.) To increase the signal-to-noise ratio, we modulate the pump and probe plus at a frequency of 10 MHz and 200 Hz, respectively, using an electro-optic modulator and optical chopper. Both pump and probe beams are focused on the sample surface, which is covered by a 3 nm Ta capping layer, with a spot size of 6 μm. The Kerr rotation of the reflected probe beam is measured by a combination of the Wollaston prism and a balanced photodetector.

## 3. Results

### 3.1. Basic Magnetic Properties

**Figure 1:**
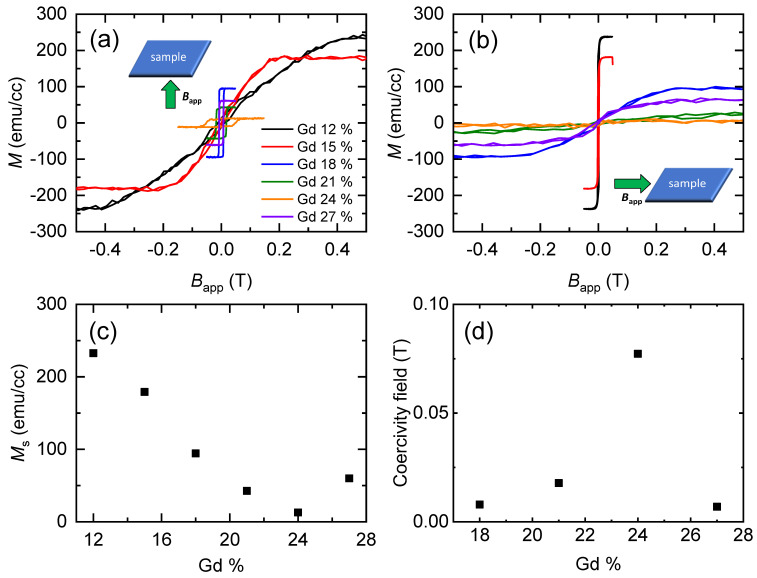
VSM results. Magnetization hysteresis of the Gd_x_Co_1-x_ alloy, x from 12% to 27%, applying the magnetic field (**a**) out-of-plane and (**b**) in-plane directions. (**c**) The saturation magnetization of the Gd_x_Co_1-x_ alloy. (**d**) The coercivity field of the out-of-plane anisotropy Gd_x_Co_1-x_ alloy.

As basic magnetic properties, we measure the hysteresis of magnetization of the GdCo alloy by applying a magnetic field along the out-of-plane and in-plane directions (Figure 1). At Gd concentrations of 18–27%, the GdCo alloy has a perpendicular magnetic anisotropy, whereas it has an in-plane magnetic anisotropy at Gd concentration of 12–15%. In particular, the net magnetization critically depends on the Gd concentration, and it becomes minimum at a Gd concentration of 24%. Therefore, Gd = 24% is close to the magnetic compensation point. In addition, the coercivity field of the perpendicularly magnetized GdCo alloy reaches a maximum at a Gd concentration of 24%. The coercivity field is often proportional to the perpendicular magnetic anisotropy; accordingly, one may expect the divergence of the magnetic anisotropy at the compensation point. However, a vanishing magnetization compensates for the divergence of the coercivity field. The saturation field along the hard axis can be used for a rough estimation of the magnetic anisotropy energy. Unfortunately, the saturation field is beyond the maximum field of VSM, and the VSM signal becomes too weak with a vanishing net magnetization near the compensation point.

### 3.2. Determination of Magnetic Anisotropy

**Figure 2:**
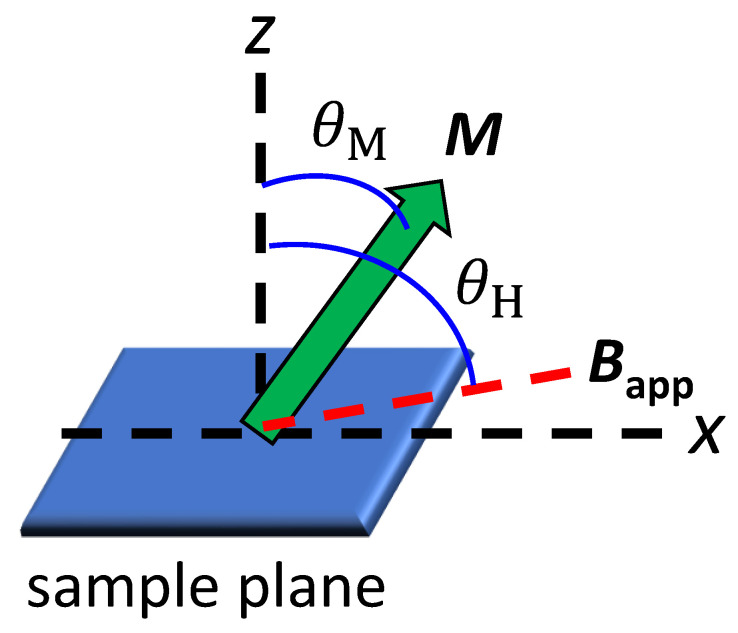
Angle definition for AHE measurements. The normal to the sample plane is defined as the *z* axis. When a magnetic field (B_app_) is applied at the angle of *θ*_H_ with respect to the *z* axis, the magnetization (*M*) tilts at an angle of *θ*_M_, which is determined by the balance between the uniaxial anisotropy energy and Zeeman energy.

To determine the uniaxial magnetic anisotropy of the GdCo alloy, we apply the GST method to the AHE reading of the normalized out-of-plane magnetization, *m*_z_ = cos *θ*_M_, where *θ*_M_ is the angle of magnetization from the sample normal (Figure 2). The AHE reading is advantageous over VSM reading in terms of signal-to-noise ratio. As the GST method analyze many *m*_z_ data with different applied fields (*B*_app_), it is more accurate than the analysis based on one data point at the saturation field. In addition, as the GST method analyze the gradual change in *m*_z_ with respect to *B*_app_, its field requirement is smaller than the saturation field.

**Figure 3:**
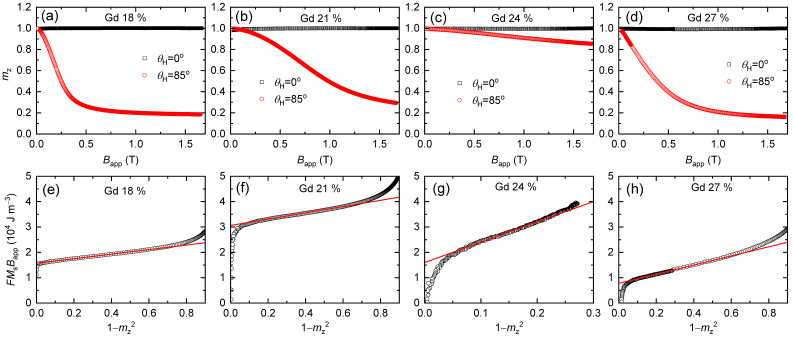
AHE results. The normalized AHE voltage (*m*_z_) of the (**a**) Gd_18_Co_82_, (**b**) Gd_21_Co_79_, (**c**) Gd_24_Co_76_, and (**d**) Gd_27_Co_73_ alloys. The magnetic field is applied with an oblique angle, *θ*_H_, with respect to the film normal direction. The black/red color corresponds to the *θ*_H_ of 0°/85°. The GST analysis of the (**e**) Gd_18_Co_82_, (**f**) Gd_21_Co_79_, (**g**) Gd_24_Co_76_, and (**h**) Gd_27_Co_73_ alloys. The black squares are the data obtained from (**a**–**d**) with *θ*_H_ of 85°. The red lines are fittings with Equation (1). The fitted values of the uniaxial anisotropy are summarized in Table 1.

We measure *m*_z_ using AHE with applying *B*_app_ at an oblique angle of *θ*_H_ = 0° or 85° (Figure 2). At *θ*_H_ of 0°, *m*_z_ is nearly independent of *B*_app_ because AHE signal, which is linearly proportional to *M*, dominates the ordinal Hall signal, which is linearly proportional to *B*_app_. With a large *θ*_H_ of 85°, *m*_z_ gradually decreases with *B*_app_ as the in-plane component of *B*_app_ tilts the magnetization (Figure 3). (*θ*_H_ should be less than 90° to have an out-of-plane component of *B*_app_, which suppresses multidomain formation.) According to the GST method, the relationship between *m*_z_ and *B*_app_ can be expressed as [22],

(1) $$2\left(K_{1}-\frac{\mu_{0}}{2}M_{S}^{2}\right)+4K_{2}\left(1-m_{z}^{2}\right)=FB_{\mathrm{app}}M_{S},$$

where *K*_1_ and *K*_2_ are the first and second-order uniaxial anisotropies, *M*_S_ is the saturation magnetization of the GdCo alloy, and *F* is given by

(2) $$F=\frac{m\sin\theta_{H}-\sqrt{1-m_{z}^{2}}\cos\theta_{H}}{m_{z}\sqrt{1-m_{z}^{2}}}.$$

Plotting $FB_{\mathrm{app}}M_{S}$ vs. $1-m_{z}^{2}$, *K*_1_ and *K*_2_ can be determined independently of the intercept and slope, respectively (Figure 3). (For the Gd = 24%, the measured range of the $1-m_{z}^{2}$ is small because the maximum B_app_ of 1.7 T is much smaller than the saturation field of $B_{\mathrm{sat}}\approx \frac{2K_{\mathrm{tot}}}{M_{S}}$, where *K*_tot_ is the total anisotropy.) The determined values of *K*_1_ and *K*_2_ with Gd concentrations of 18%, 21%, 24%, and 27% are summarized in Table 1. The maximum *K*_tot_ = *K*_1_ + *K*_2_ of 2.8 × 10^4^ J m^−3^ is obtained at the compensation point of Gd = 24%. Previously reported values of *K*_tot_ of the GdCo alloy are in the range of 10^4^ J m^−3^, depending on the Gd concentration and deposition method [1,2,20]. We note that the anisotropy energy of the GdCo alloy is about two orders of magnitude smaller than that of FePt, a well-known ferromagnet for strong magnetic anisotropy [22,23]. Such a low magnetic anisotropy of the GdCo alloy limits the application to memory devices. Interestingly, *K*_2_ becomes larger than *K*_1_ at the compensation point. This observation is surprising because *K*_1_ is usually much larger than *K*_2_ for typical ferromagnets [22,23]. To understand the physical origin for the strong enhancement of *K*_2_ at the compensation point, further theoretical and experimental works are required.

### 3.3. Determination of Damping Constant

**Figure 4:**
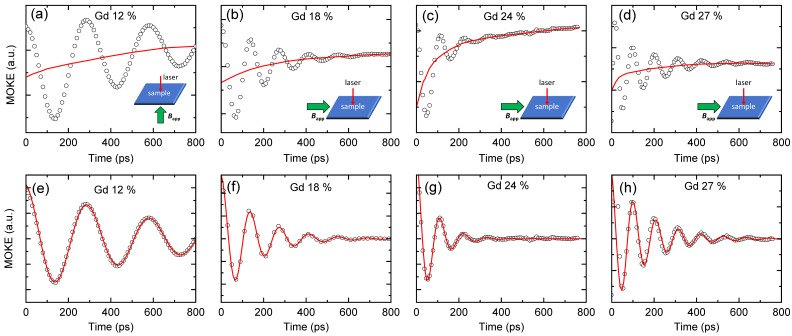
TRMOKE results. The magnetization dynamics triggered by a pump pulse in the (**a**) Gd_12_Co_88_, (**b**) Gd_18_Co_72_, (**c**) Gd_24_Co_76_, and (d) Gd_27_Co_73_ alloys. A magnetic field of 0.5 T/0.3 T is applied along the z/x direction for (**a**)/(**b**–**d**). The black circles are the measure data. The red lines are the background demagnetization signal. The extracted precessional motion in the (**e**) Gd_12_Co_88_, (**f**) Gd_18_Co_72_, (**g**) Gd_24_Co_76_, and (**h**) Gd_27_Co_73_ alloys. The black circles are obtained by subtracting the demagnetization background from the raw data of (**a**–**d**). The red lines are fittings by damped cosine function of cos(2πft)×exp(−t/τ). The fitted *f* values are 3.4, 7.2, 8.9, and 9.4 GHz for (**e**–**h**), respectively. The fitted *τ* values are 600, 160, 80, and 160 ps for (**e**–**h**), respectively.

To determine damping constants of the GdCo alloy, we measure the magnetization dynamics using TRMOKE. We use four samples with Gd concentrations of 12%, 18%, 24%, and 27%. The Gd = 12% has in-plane anisotropy, whereas others have out-of-plane anisotropy. To trigger the magnetization precession, we need to apply *B*_app_ along the *z*/*x* direction for the in-plane/out-of-plane anisotropy sample. We apply a *B*_app_ of 0.3 T along the *x* direction for the Gd_18_Co_82_, Gd_24_Co_76_, and Gd_27_Co_73_ samples and *B*_app_ of 0.5 T along the *z* direction for the Gd_12_Co_88_ sample. The magnitude of *B*_app_ is chosen to be larger than the saturation field along the hard axis, so that the initial magnetization aligns along the *x* or *z* direction (Figure 1). (This is not the case for the Gd = 24%, in which the saturation field is much larger than *B*_app_ of 0.3 T.) Such an alignment makes the damping analysis to be simple. The equilibrium direction of magnetization is determined by the balance between the uniaxial anisotropy, demagnetization field, and external field. When a pump pulse induces an ultrafast demagnetization via sudden heating, the balance is suddenly disturbed, and a precessional motion of magnetization is triggered [25]. Indeed, the TRMOKE shows a precessional motion on top of an ultrafast demagnetization (Figure 4). We separate the precessional motion by subtracting the demagnetization background signal from the raw data. The precessional motion can be described by the Landau–Lifshitz–Gilbert equation based on the mean-field model [26,27,28,29],

(3) $$\frac{d}{dt}{\vec{M}}_{\mathrm{eff}}=-\gamma_{\mathrm{eff}}{\vec{M}}_{\mathrm{eff}}\times {\vec{H}}_{\mathrm{eff}}+\frac{\alpha_{\mathrm{eff}}}{M_{\mathrm{eff}}}\left({\vec{M}}_{\mathrm{eff}}\times \frac{d}{dt}{\vec{M}}_{\mathrm{eff}}\right),$$

where *M*_eff_ is the net magnetization of the GdCo alloy, *γ*_eff_ is the effective gyromagnetic ratio of the GdCo alloy, *H*_eff_ is the effective field combining the exchange field, anisotropy field, demagnetization field, and external field, and *α*_eff_ is the effective damping of the GdCo alloy. Alternatively, *α*_eff_ can be obtained as $\alpha_{\mathrm{eff}}=1/\left(2\pi f\tau \right)$, where *f* is the precession frequency, and *τ* is the relaxation time of the damped cosine function of cos(2πft)×exp(−t/τ).

**Figure 5:**
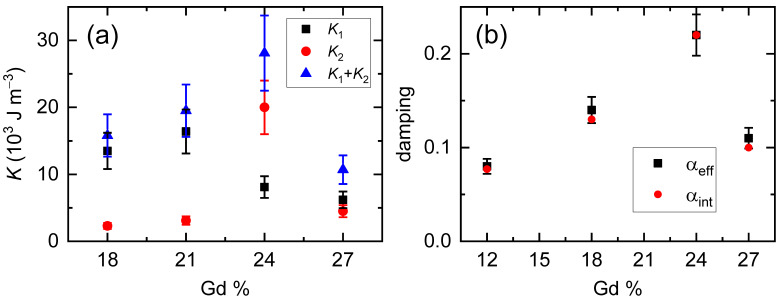
Summary of the uniaxial anisotropy (*K*) and damping constant (*α*). (**a**) The *K* values of the GdCo alloy. The black square/red circle/blue triangle corresponds to the first order (*K*_1_)/second order (*K*_2_)/total (*K*_1_ + *K*_2_) anisotropy. The error range in the *K*_1_ and *K*_2_ determination is 20% considering the 10% uncertainty in *M*_S_ of VSM measurements and ±1° error in *θ*_H_ of AHE measurements. (**b**) The *α* values of the GdCo alloy. The black square/red circle corresponds to the effective damping (*α*_eff_)/intrinsic damping (*α*_int_). The *α*_eff_ is obtained from the fitting of the precession motion. The *α*_int_ is obtained using Equation (4) for the Gd = 12%, 18%, and 27%. For the Gd = 24%, we assume *α*_int_ is the same as *α*_eff_. The error range in the *α*_eff_ determination is 10% considering the fitting uncertainties of *f* and *τ*.

We summarize the determined values of *K*_tot_ (*K*_1_ + *K*_2_) and *α*_eff_ in Figure 5. Both *K*_tot_ and *α*_eff_ maximize at the compensation point of Gd = 24%. Here, *α*_eff_ is not the intrinsic parameter, but depends on *B*_app_. For the Gd_18_Co_82_ and Gd_27_Co_73_ alloys, when magnetization aligns nearly to the *x* direction by *B*_app_, the intrinsic damping (*α*_int_) is related to *α*_eff_ as [30],

(4) $$\alpha_{\mathrm{int}}\approx \alpha_{\mathrm{eff}}\frac{2\sqrt{B_{1}B_{2}}}{B_{1}+B_{2}},$$

where *B*_1_ = *B*_app_ + *B*_K_, *B*_2_ = *B*_app_, and *B*_K_ = (2*K*_1_ − *μ*_0_*M*_S_ + 4*K*_2_)/*M*_S_. For the Gd_24_Co_76_ alloy, magnetization aligns nearly to the *z* direction by strong *B*_ani_, then α_int_≈α_eff_. The variation of *α*_eff_ of the Gd_24_Co_76_ alloy with different *B*_app_ is shown in Appendix A. Using the *B*_K_ information in Table 1, we show that α_int_ also maximize at Gd = 24% with a peak value of 0.22. Our result of *α* = 0.22 is consistent with previous reports of 0.2–0.3 of the GdCo and GdFeCo alloys from the ferromagnetic resonance and TRMOKE measurements [26,27,28]. However, much low *α* of 0.007 of the GdFeCo alloy was reported from the measurement of the domain wall motion [31]. Further studies are required to resolve this discrepancy between measurement techniques.

Note that the pump pulse induces a significant temperature rise in the sample. Considering $\Delta T\approx F_{\mathrm{in}}A_{\mathrm{tot}}/C_{V}d_{\mathrm{tot}}$, where *F*_in_ is the incident fluence of the pump of 12 J m^−2^, *A*_tot_ is the absorption coefficient by the total metal layers of ≈ 0.3, *C*_V_ is the typical heat capacity of metals of 3 × 10^6^ J m^−3^ K^−1^, and *d*_tot_ is the total thickness of metal layers of 21 nm, the temperature rise of the sample would be approximately 60 K (ignoring the slow heat transfer to the substrate). Because the alloy concentration for the magnetic compensation depends on temperature, the Gd_24_Co_76_ alloy may not be the magnetic compensation point during TRMOKE. We claim that the increase in *α*_eff_ is caused by the angular momentum compensation between the Gd and Co sublattices. Such enhancement of *α*_eff_ near the compensation point has been previously reported for the GdFeCo and GdCo alloys, and the mean-field model was used to explain the physical origin [26,27,28]. According to the mean-field model, *α*_eff_ of the GdCo alloy is expressed as [29],

(5) $$\alpha_{\mathrm{eff}}=\frac{\alpha_{\mathrm{Co}}M_{\mathrm{Co}}/\gamma_{\mathrm{Co}}+\alpha_{\mathrm{Gd}}M_{\mathrm{Gd}}/\gamma_{\mathrm{Co}}}{M_{\mathrm{Co}}/\gamma_{\mathrm{Co}}-M_{\mathrm{Gd}}/\gamma_{\mathrm{Co}}},$$

where α_Co_/α_Gd_ is the damping constant of the Co/Gd sublattice, *M*_Co_/*M*_Gd_ is the magnetization of the Co/Gd sublattice, and *γ*_Co_/*γ*_Gd_ is the gyromagnetic ratio of the Co/Gd sublattice. Accordingly, *α*_eff_ diverges at the compensation of the angular momentum, M/γ. Typically, the angular momentum compensation temperature is 50–100 K higher than the magnetization compensation temperature [13,15,27].

## 4. Discussion

We investigate the magnetic anisotropy energy and damping constant of the GdCo alloy. By applying the GST method to the AHE measurements, we determine the uniaxial anisotropy energy of 2.8 × 10^4^ J m^−3^ at the compensation point, Gd concentration of 24%. Surprisingly, we find that the second-order uniaxial anisotropy becomes larger than the first-order one at the compensation point. We expect that this anisotropy inversion is related to the magnetization compensation. Measuring the magnetization dynamics using TRMOKE, we determine the damping constant. An enhanced damping constant of 0.22 is observed at the compensation point. This enhancement is consistent with previous reports and can be understood by the angular momentum compensation.

## Figures and Tables

**Table 1:** The first-order (*K*_1_) and second-order (*K*_2_) uniaxial anisotropy of the GdCo alloy, with the Gd concentration of 18%, 21%, 24%, and 27%. The *K*_1_^eff^ and *K*_2_ values are determined from the linear fitting of Figure 2e–h. The *K*_1_ values are obtained from *K*_1_^eff^ by *K*_1_ = *K*_1_^eff^ + *μ*_0_*M*^2^/2.

Colume Heading	Gd_18_Co_82_	Gd_21_Co_79_	Gd_24_Co_76_	Gd_27_Co_73_
*K*_1_^eff^ (J m^−3^)	7.9 × 10^3^	15.3 × 10^3^	8 × 10^3^	3.9 × 10^3^
*μ*_0_*M*^2^/2 (J m^−3^)	5.6 × 10^3^	1.1 × 10^3^	0.1 × 10^3^	2.3 × 10^3^
*K*_1_ (J m^−3^)	13.5 × 10^3^	16.4 × 10^3^	8.1 × 10^3^	6.2 × 10^3^
*K*_2_ (J m^−3^)	2.3 × 10^3^	3.1 × 10^3^	20 × 10^3^	4.5 × 10^3^
*K*_1_ + *K*_2_ (J m^−3^)	15.8 × 10^3^	19.5 × 10^3^	28.1 × 10^3^	10.7 × 10^3^

**Figure A1:**
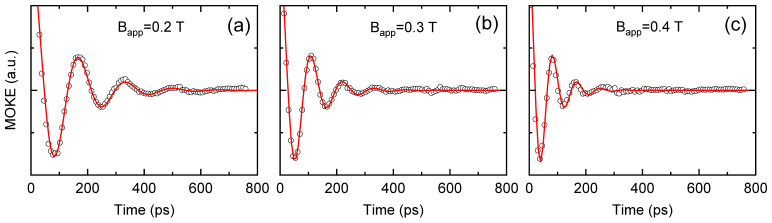
The *B*_app_ dependence on *α*_eff_. The precessional motion in the Gd_24_Co_76_ alloys with *B*_app_ of (**a**) 0.2, (**b**) 0.3, and (**c**) 0.4 T along the *x*-direction. The black circles are obtained by subtracting the demagnetization background from the raw data of TRMOKE. The red lines are fittings by damped cosine function of cos(2πft)×exp(−t/τ). The fitted *f* values are 6.0, 8.9, and 11.8 GHz for (**a**–**c**), respectively. The fitted *τ* values are 120, 80, and 60 ps for (**a**–**c**), respectively.

## Data Availability

Derived data supporting the findings of this study are available from the corresponding author (G.-M.C.) on request.

## Appendix A

We measure the magnetization precession of the Gd_24_Co_76_ alloy with three different *B*_app_ of 0.2, 0.3, and 0.4 T. From the fittings with a damped cosine function, we determine the same *α*_eff_ of 0.22 ± 0.02 for all three conditions.
